# Effects of Biochar Amendment on Potassium Supply Capacity and Potassium Accumulation in Soybean Across Diverse Soils

**DOI:** 10.3390/plants14131959

**Published:** 2025-06-26

**Authors:** Liqun Xiu, Yuanyuan Sun, Xiaori Han

**Affiliations:** 1 Postdoctoral Station of Agricultural Resources and Environment, Land and Environment College, Shenyang Agricultural University, Shenyang 110866, China; xlq@syau.edu.cn; 2 Key Laboratory of Biochar and Soil Improvement, Ministry of Agriculture and Rural Affairs, Shenyang 110866, China; 2020200076@stu.syau.edu.cn; 3 National Biochar Institute, Shenyang Agricultural University, Shenyang 110866, China; 4 Agronomy College, Shenyang Agricultural University, Shenyang 110866, China

**Keywords:** biochar, soil potassium supply capacity, crop potassium accumulation, Q/I curve, potassium coordination theory

## Abstract

Biochar enhances soil available potassium and plant uptake, yet its effects on soil potassium supply capacity and crop potassium accumulation require clarification. This study used a pot experiment with three soil types (albic, brown, and sandy soils) and four biochar application rates (0, 10, 20, and 30 g·kg^−1^) to investigate potassium supply capacity and soybean potassium accumulation using the potassium site coordination theory and Q/I curve analysis. The results showed that biochar significantly increased the available potassium content in soil. At the highest biochar application rate (30 g·kg^−1^), the available potassium in the albic, sandy, and brown soils increased by 24.84%, 60.90%, and 24.84%, respectively, compared to the control. The biochar boosted the instantaneous potassium supply (elevated AR_0_ and ΔK values) through direct water-soluble potassium input. However, the potential potassium supply capacity (PBC) varied by soil type: the PBC increased in the brown soil at low application rates but decreased in the albic and sandy soils with higher rates. The biochar enhanced soybean potassium accumulation through two pathways: the direct enrichment of soil potassium pools and the indirect improvement in soil properties to promote biomass accumulation. These findings provide theoretical insights for optimizing biochar use in agriculture to maximize potassium availability and crop efficiency.

## 1. Introduction

Potassium is one of the three essential nutrients for plants, constituting 2–10% of plants’ dry weight [1]. It exists predominantly as potassium ions (K^+^), which are widely distributed across various plant organs and represent the most abundant monovalent cation in plants. These ions play a critical role in crop growth and development [2]. In terms of abundance, potassium ranks as the seventh most common element in the Earth’s crust, accounting for 2.1–2.3% of its total mass [3]. However, despite the relatively high potassium content in soils, 96–98% is locked in mineral forms that plants cannot readily utilize [4]. In China, intensive farming practices have led to continuous potassium depletion, resulting in a negative potassium balance in agricultural soils [5]. This imbalance has significantly degraded the soil quality [6]. Consequently, potassium deficiency remains a persistent challenge in Chinese agriculture, with scientific fertilization strategies being critical to mitigating the effects of soil potassium deficits [7]. China faces a scarcity of potassium fertilizer resources, having imported over 63 million tons of K_2_O since the 1970s, leading to high dependency on external supplies [8].

Developing diverse potassium supplementation methods is essential to ensuring potassium availability in farmlands [9]. In recent years, biochar application has gained significant attention due to its agricultural potential [10]. Biochar, a solid product formed through the pyrolysis of agricultural and forestry waste under high-temperature, oxygen-limited conditions [11], releases potassium from biomass during production, transforming it into crop-available forms [12]. Research indicates that biochar not only directly supplements soil potassium but also enhances its availability by modifying the structure of active potassium pools [12]. Previous studies have identified multiple potassium forms in biochar, including water-soluble potassium, exchangeable potassium, non-exchangeable potassium, and unavailable potassium, with non-water-soluble forms constituting over 50% of the total potassium content [13,14].

Evaluating biochar’s effect on soil potassium supply capacity requires a multidimensional approach [14]. In terms of abundance of the different potassium forms, the potassium site coordination theory explains that most soil potassium is adsorbed and fixed by clay minerals rather than existing as ionic salts [15]. These adsorption sites are classified into three types: p-sites on clay particle surfaces, e-sites at mineral crystal edges, and i-sites within mineral interlayers [16,17]. From a dynamic equilibrium perspective, the potassium quantity–intensity (Q/I) curve quantifies the relationship between potassium ion intensity (I) in the soil solution and solid-phase potassium capacity (Q), providing a systematic measure of soil potassium supply capacity [18,19]. Cations such as Ca^2+^ and Mg^2+^ in the soil solution influence potassium availability through competitive adsorption, so the aK/(aCa + aMg)^1^/^2^ ratio is commonly used to represent the potassium supply intensity (ΔR) [20,21]. Furthermore, different soils vary in their ability to sustain a specific ΔR value, necessitating a comprehensive evaluation that incorporates changes in exchangeable potassium (ΔK, the capacity factor Q) [20,21].

Biochar’s regulatory effect on soil potassium ultimately influences crop potassium accumulation. Biochar affects plant potassium uptake through both direct and indirect mechanisms [22]. The direct effect involves increasing the readily available potassium content in the soil, thereby enhancing the potassium supply efficiency, promoting plant potassium absorption and elevating potassium levels in plants [23]. The indirect effect from biochar improves overall plant development [24] and, in turn, enhances potassium uptake due to increased plant vigor [25]. However, the specific contributions of these two mechanisms to crop potassium accumulation remain unclear.

To comprehensively assess biochar’s role in regulating soil potassium dynamics, this study applied varying amounts of biochar to different soil types and investigated its effects on soil potassium availability, potassium site distribution, and Q/I curves to elucidate how biochar modulates soil potassium supply capacity. Using soybean as a test crop, the study further explored biochar’s impact on crop potassium accumulation. By examining the changes in the soil potassium supply capacity, as well as the potassium content and dry matter accumulation in different soybean organs, this research aimed to clarify biochar’s influence on soil potassium dynamics, soybean growth, and potassium uptake across diverse soils. These findings will provide a scientific foundation for effectively utilizing the potassium in biochar.

## 2. Results

### 2.1. Soil Properties

The application of biochar improved the properties of three types of soil: albic, sandy, and brown soils. The biochar was found to enhance not only the levels of nitrogen (N) and phosphorus (P) (Table 1) but also potassium in the soil (Table 2). Additionally, the porous structure of the biochar improved the physical characteristics of the soils by reducing the bulk density when 20 and 30 g/kg of biochar were added. The alkaline nature of the biochar increased the soil pH in the acidic and neutral soils (albic and brown soils), while it decreased the pH in the alkaline sandy soil when 10 and 20 g/kg of biochar were added.

### 2.2. Soil Potassium Availability

As shown in Table 2, the biochar application significantly enhanced the available potassium content in all soil types in a dose-dependent manner. The maximum application treatments (A3, S3, and B3) exhibited 24.84%, 60.90%, and 24.84% increases in the available potassium content compared to the control groups, respectively. This enhancement was due to the biochar-induced improvements in the amounts of the various potassium forms.

The biochar application substantially elevated the water-soluble potassium levels, with the treatment efficacy positively correlating with the application rate. The A3, S3, and B3 treatments demonstrated remarkable increases of 82.30%, 38.02%, and 124.26% in the water-soluble potassium content relative to the controls.

Furthermore, the biochar amendments effectively enhanced both the soluble and insoluble potassium fractions. Exchangeable potassium levels showed particularly pronounced improvements, with the A3, S3, and B3 treatments achieving 58.14%, 420.64%, and 50.23% increases compared to the control groups. Simultaneously, the non-exchangeable potassium content also increased by 16.62%, 37.13%, and 37.13%.

### 2.3. Potassium Adsorption State in Soil

In all three types of soil, the biochar increased the amount of i-site potassium in the soil in an application rate-dependent manner (Table 3). The treatments with the highest biochar application rate (A3, S3, and B3) increased the amount of i-site potassium by 20.07%, 80.92%, and 36.40%, respectively, compared to the control.

In both the albic and brown soils, the amount of p-site potassium increased with increasing biochar application rate. The highest biochar application rate increased the amount of p-site potassium by 62.49% and 52.38%, respectively, compared to the control. The biochar only increased the levels of e-site potassium in the brown soil, with Z3 increasing the levels by 52.03% compared to the control.

### 2.4. Soil Potassium Q/I Curve and Parameters

**Figure 1:**
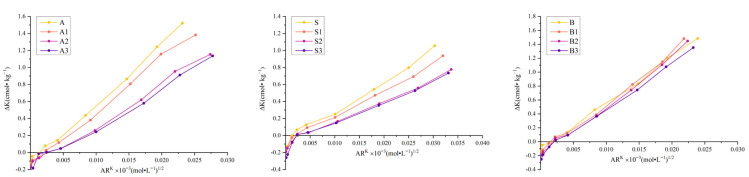
Effect of different application rates of biochar (0, 10, 20, and 30 g/kg) on the soil potassium Q/I curve.

The main impact of the biochar on the instantaneous potassium supply capacity of the soils was an increase in the AR0 and ΔK content (Figure 1 and Table 4). The soil potassium balance activity ratio (AR0) is an indicator of soil potassium availability, generally referred to as the soil potassium intensity index (I). The addition of the biochar significantly improved this index in a dose-dependent manner. ΔK is the content of active potassium in the soil, which was increased by the addition of the biochar.

The impact of the biochar on PBC (Table 4), which is the potential potassium supply capacity, differed depending on the soil type. For the albic and sandy soils, the PBC decreased with increasing biochar application rate. However, the brown soil showed an increase in PBC with low and moderate levels of biochar, with B2 and B1 increasing it by 6.53% and 3.65%, respectively, compared to the control. When the biochar application rate was 30g/kg, the PBC decreased.

### 2.5. Potassium Content in Soybean Plants

**Figure 2:**
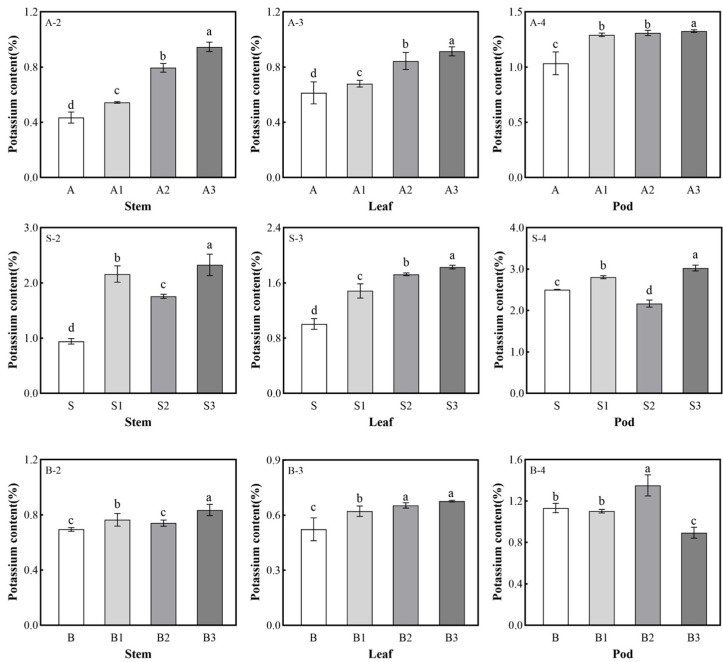
Effect of different application rates of biochar (0, 10, 20, and 30 g/kg) on potassium content in soybean plants. The different lowercase letters in the same graph indicate significant differences (*p* < 0.05) between the different amounts of biochar applied to the same soil type.

Potassium is a key element for plant growth, and biochar can promote the absorption of potassium by soybean plants (Figure 2). When added to all three types of soils, the biochar promoted potassium absorption in soybean stems and leaves, and the potassium content was higher than in the control. The highest biochar application rate induced the highest potassium content in the stems. In the white soil, the A3 treatment resulted in the highest potassium content, which was 117.83% higher than the control, while S3 increased the potassium content in the soybean stems by 146.64% compared to the control. The application of treatment B3 to the brown soil increased the potassium content by 20.25% compared to the control. In the leaves, the biochar treatment significantly increased the potassium content in an application rate-dependent manner. The application of 30 g/kg of biochar to the three soils (A3, S3, and B3) resulted in potassium contents of 49.04%, 82.01%, and 29.31%, respectively, in the leaves.

As shown in Figure 2, the application of the biochar to the white soil increased the potassium content in the soybean pods in a dose-dependent manner. The potassium content in the A3 soybean pods increased by 28.08% compared to the control. In the sandy soil, when the biochar application rates were 10 g/kg and 30 g/kg, the potassium contents in the pods were 12.16% and 21.06% higher than the control, respectively. The application of biochar treatment B2 to the brown soil increased the potassium content in the pods by 19.31% compared to the control.

### 2.6. Dry Matter Accumulation in Soybean Plants

**Figure 3:**
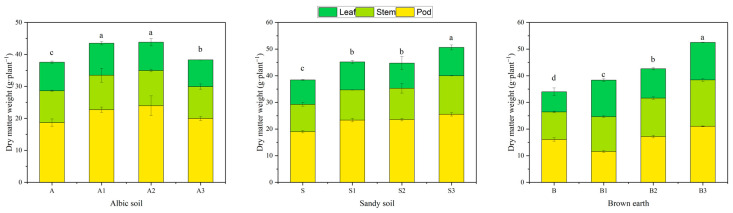
Effect of different application rates of biochar (0, 10, 20, and 30 g/kg) on dry matter accumulation in soybean plants. The different lowercase letters in the same graph indicate significant differences (*p* < 0.05) in dry matter accumulation in soybean plants grown in the same soil type but treated with different amounts of biochar.

The biochar promoted the growth of soybeans but the growth during the pod-setting period varied depending on the amount of biochar and the type of soil. In the albic soil, the promoting effect of the biochar was highest with a low to medium biochar dose. The A1 and A2 biochar treatments increased aboveground growth and dry matter accumulation by 15.76% and 16.67%, respectively, compared to the control (Figure 3). The application of the biochar treatment mainly increased the dry weight of pods and stems. The biochar increased the dry matter accumulation in large pods, with A2 and A3 increasing it by 21.75% and 28.39%, respectively, compared to the control.

In the brown and sandy soils, soybean plant growth increased with increasing biochar application, with S3 and B3 increasing growth by 31.78% and 54.49%, respectively. For medium and low biochar contents, the main increase in dry matter was in the pod weight. Using the high biochar treatments (S3 and B3), the dry matter weight of the stem increased by 41.54% and 68.25%, respectively. In the brown soil, the high biochar treatment also increased the dry matter weight of the leaves by 86.02%.

### 2.7. Accumulation of Potassium in Soybean Plants

In all three types of soil, the biochar increased the total potassium accumulation in soybean plants in an application rate-dependent manner (Table 5). Compared with the control, the highest potassium accumulation levels were observed in the A2, S3, and B3 treatments, which increased the levels by 58.73%, 100.15%, and 46.49%, respectively. The effect of the biochar on potassium accumulation in the different parts of the soybean plants varied depending on the amount of biochar applied. The biochar promoted the accumulation of potassium in both the soybean stems and leaves, which correlated with the biochar application rate. In both the albic and brown soils, the potassium accumulation in large bean pods showed a trend of first increasing and then decreasing with increasing biochar application rate, with the highest accumulation observed with 20g/kg. This indicates that a high biochar content has a certain inhibitory effect on pod formation, which affects the final yield.

### 2.8. Regulation of Potassium Accumulation in Soybean Plants by Biochar

**Figure 4:**
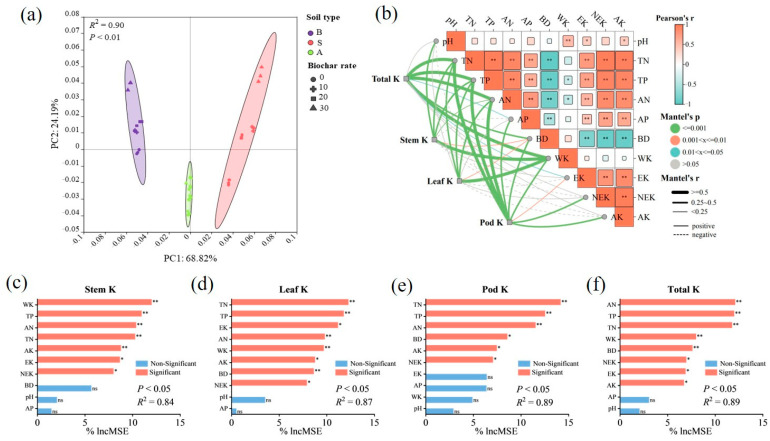
Factor analysis results. (**a**) PCA; (**b**) Mantel test and Spearman correlation; (**c**–**f**) identification of factors influencing potassium uptake in soybean plants based on random forest analysis. *: 0.01 < *p* < 0.05; **: 0.001 < *p* < 0.01; ns: Non-Significant.

The PCA results indicate that the effects of the different soil types and amounts of biochar on the soil physicochemical properties and potassium components could be divided into two characteristic vectors, which explain a total of 93.01% of the variance, with PC1 accounting for 68.82% and PC2 accounting for 24.19% (Figure 4). The soil type was found to be the main factor (*p* < 0.01). The correlation analysis found that the total nitrogen, total phosphorus, active nitrogen, and phosphorus levels were significantly positively correlated with the exchangeable potassium, non-exchangeable potassium, and available potassium levels, while the bulk density was significantly negatively correlated with them. The correlation between water-soluble potassium levels and soil properties was relatively low. The Mantel test analysis and random forest factor evaluation results indicated that the potassium accumulation in different parts of the plants was regulated by different factors. Although these factors differed between different parts of the plant, they all had direct effects on the soil potassium content and indirect effects on the soil properties.

**Figure 5:**
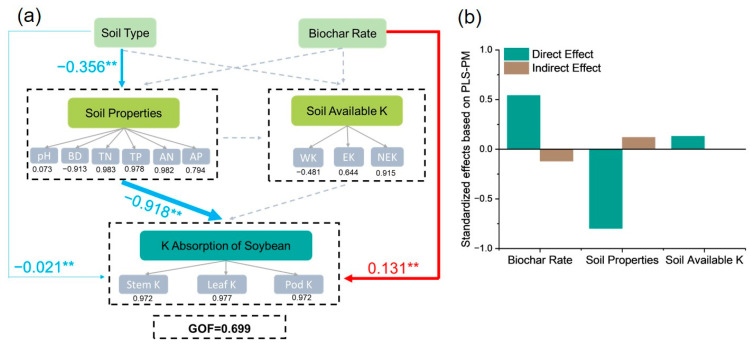
Partial least squares path analysis results: (**a**) path analysis; (**b**) direct and indirect effects of various factors on plant potassium bioavailability. **: 0.001 < *p* < 0.01.

A pathway analysis was conducted using PLS-PM to elucidate how biochar regulates soybean potassium accumulation pathways, and the GOF = 0.699 model was found to fit well (Figure 5). The PLS-PM fitting results indicate that biochar can affect soybean potassium uptake by directly increasing the soil potassium availability and indirectly altering the soil properties (β = 0.131, *p* < 0.05).

### 2.9. Yield

The biochar increased the yield of the soybean plants grown in the three types of soils (Table 6). However, the changes in the different yield components in the three types of soils were different. In the albic soil, the biochar increased the final soybean yield by increasing the weight and number of main stems and branches. An increase in biochar content increased the branch grain weight; the A3 treatment increased the branch grain weight by 100.00% compared to the control. In the sandy and brown soils, the highest increase in carbon application was observed at a biochar application rate of 10 g/kg.

## 3. Discussion

A large amount of potassium in soil exists as ineffective potassium in soil minerals [4]. Although biochar contains a large amount of effective potassium, its impact on total potassium levels when added to soil is limited. Therefore, biochar mainly affects the available potassium in soil. After biochar is applied to soil, it adds a large amount of active potassium sources to the soil. In this study, the effective potassium content in all three types of soil increased after the biochar application in a dose-dependent manner. Previous studies have mostly explored the regulatory effect of biochar on soil-available potassium by focusing on the input of water-soluble potassium from biochar. In our previous research, we demonstrated the presence of non-water-soluble potassium in biochar, indicating that the regulatory effect of biochar on soil potassium is not limited to the input of the potassium carried by the biochar. Therefore, this study explored how biochar affects the soil potassium supply from the perspective of the potassium supply capacity and analyzed the impact of biochar application on soybean potassium accumulation.

### 3.1. Potassium Availability

The application of the biochar to the three types of soil increased the available potassium content in the soils, but the increases in the different forms of potassium were different. The application of the biochar to the soils increased the water-soluble potassium content in the soil in a dose-dependent manner. This increase was due to the release of water-soluble potassium from the biochar. Biochar contains a large amount of water-soluble potassium, which exists in the form of ionic salts. Therefore, when applied to soil, it will have a strong “potassium fertilizer effect” [10].

The formation of exchangeable and non-exchangeable potassium essentially depends on the binding state between potassium elements and the soil [26]. The main sources of exchangeable potassium are p-site potassium and e-site potassium. p-sites are located on the outer surface of clay particles and have a relatively weak adsorption affinity for potassium ions. e-sites are distributed at the edge or wedge-shaped zone of particles and have a stronger ability to bond potassium through electrostatic interactions than p-sites [16]. It is worth noting that there was no significant increase in the e-site potassium content in the soils. This indicates that the effect of biochar on exchangeable potassium is mainly due to the increase in p-site potassium. Although the content of both e-site and p-site potassium in biochar is the same, they do not show the same effects in soil, which may be due to the adsorption of potassium by biochar. Biochar may fix more potassium at e-sites through adsorption, becoming i-site potassium and thus non-exchangeable potassium. This adsorption effect was also confirmed for the non-exchangeable potassium.

The main source of non-exchangeable potassium is the i-site potassium in biochar. Non-exchangeable potassium can also be influenced by more complex factors, which differ depending on the soil type. The impact of the biochar on i-site potassium was weaker than the effects on the other two types of potassium; the effect on i-site potassium was mainly related to the continuous potassium supply capacity of the biochar. The common interaction point between them is the input effect of biochar on i-site potassium. i-sites are special positions that only exist in the lattice of 2:1-type clay minerals and exhibit a strong adsorption affinity for potassium ions (K^+^) [27]. i-site potassium is mainly interlayer potassium, and it is difficult for biochar to affect the interlayer composition of soil. Therefore, the increase in i-site potassium was primarily due to the input effect of biochar. In addition, biochar introduces a large amount of available potassium into the soil. Different forms of potassium in the soil are transformed into each other in dynamic equilibrium. When the exchangeable and water-soluble potassium contents increase, they naturally transform into slow-release potassium, which is stored in the interlayer and slowly released [28].

### 3.2. Potassium Supply Capacity

The effectiveness of potassium is dependent on the seasonal potassium supply capacity. The impact of biochar on soil effectiveness is not only due to the instantaneous increase in the potassium supply capacity but also the effects on the sustained potassium supply capacity. The potassium supply capacity of biochar determines its effectiveness, and attention should also be paid to the sustained potassium supply capacity. The different forms of potassium in soil after the application of biochar are derived from the changes in the soil potassium supply capacity caused by the biochar, as well as the distribution and different forms of potassium. This study found that the application of biochar acted through the same regulatory pathway in the three different types of soil, indicating that biochar has similar regulatory effects on different soils.

Through the analysis of the Q/I curve, it was found that the biochar increased the instantaneous potassium supply capacity of the soils but it reduced the potential potassium supply capacity in the albic and sandy soils. This change confirmed the regulatory mechanism of biochar on soil potassium dynamics. The potassium in biochar is mainly water-soluble potassium and bound potassium [12], of which, the water-soluble potassium can directly supplement the active potassium in the soil, thereby improving the instantaneous potassium supply capacity [29]. However, it is difficult for potassium to precipitate into the e-site potassium in soil, as the storage of e-site potassium determines its ability to release or fix potassium during fluctuations in the potassium concentration. The more potassium in e-sites, the stronger the buffering capacity of the soil against changes in the potassium concentration and the higher the PBC value. When the potassium content in e-sites is low and there is a slight change in the potassium concentration, less potassium can be released or fixed, resulting in a smaller PBC.

In addition, the reduction in soil PBC by biochar may also be attributed to the adsorption of potassium onto soil particles [30]. Biochar is likely to convert potassium into more stable i-site potassium through adsorption, without adhering to the soil surface. In fact, it may also be converted into potassium, which is more stable than i-site potassium. By comparing the changes in the different forms of potassium in the three soils, we found that with the same input of biochar, the changes in the different forms of potassium in the three soils differed. In the albic and sandy soils, the potassium was likely to become more stable than i-site potassium. The i-site potassium content in the brown soil was much higher than in the other two types of soil. When the biochar was applied, the adsorption capacity of the biochar for potassium did not increase as the concentration increased, and no additional potassium was replaced. Therefore, biochar not only increases the supply of water-soluble potassium in soil, producing a potassium fertilizer effect, but it also increases the supply of fixed potassium.

### 3.3. Potassium Accumulation

The factor analysis and pathway analysis found that biochar can regulate crop potassium accumulation through direct and indirect effects. The direct effect stems from the improvement in the soil potassium supply capacity, while the indirect effect stems from the soil improvement effect.

The application of biochar can increase the available potassium pools in soil and enhance the potassium sources that roots can absorb [31]. The increase in absorbable potassium sources promotes the uptake of potassium by the root system [32]. The potassium content in the stems and leaves of soybean plants grown in the biochar-treated soils increased with increasing biochar application rate. The potassium absorbed by the root system is mainly transported through the xylem and phloem, so the increase in the stem potassium content indicates that the biochar application enhanced the plants’ potassium transport capacity [33].

The enhancement of the potassium transport capacity of soybean plants was partly due to the increase in the available potassium reserves in the soil, and partly due to the promotion of growth by the biochar, which indirectly increases the demand for potassium in soybean plants since potassium plays a key role in plant metabolism and growth [34]. The increase in the potassium content in the leaves supports this inference. In leaves, growth, stomatal maintenance, and other processes require potassium, making leaves a potassium-demanding organ [35]. The increase in the potassium content indicates that the biochar application improved the soybean plants’ utilization of potassium. The pods of soybeans are another potassium-demanding organ [36], and their potassium content varied depending on the soil type and biochar application rate. For the brown soil, the application of biochar reduced the potassium content of soybean pods. This decrease in potassium content was due to the reduced absorption of potassium by the roots since the potassium content in the brown soil treated with biochar was lower than that of the control. This decrease in potassium uptake may be due to the increase in soil pH, which reduces the efficiency of crop roots in obtaining potassium [37].

### 3.4. Crop Yield

The improvement in soil properties by the biochar was mainly reflected in the increased crop yield [38]. The soybean yield from the biochar-treated soils was superior to that of the control. The regulatory effect of biochar on the yield components varied for the different soils. For the relatively viscous albic soil, the addition of the biochar promoted the growth of both the main stem and lateral branches. This manifested as an increase in the grain weight of the soybeans with increasing biochar application rate. In the sandy soil, the yield of the biochar-treated plots was higher than the control, with the highest yield obtained with the S1 biochar application rate. At higher biochar application rates (S2 and S3), the number and weight of the soybean grains in the main stem and branches were lower compared to those of S1, leading to reduced yields. In the brown soil, the high biochar application rate showed an increased biomass during crop growth, but the final yield was significantly lower compared to that of the other application rates. In the later growth stage, an increased biochar input did not transfer more nutrients to the soybean seeds.

## 4. Materials and Methods

### 4.1. Experimental Materials

The soils used in this experiment were albic soil, brown soil, and sandy soil. According to the WRB (World Reference Base for Soil Resources) classification, they belong to Albeluvisols, Arenosols, and Luvisols, respectively. The biochar material consisted of corn stalk biochar granules produced by the Liaoning Jinhefu Agricultural Development Co., Ltd. (Xiuyan, Liaoning, China), which were carbonized at 400–450 °C. The biochar had a pH of 8.56 and contained 50.60% C, 1.4% N, 0.46% P, and 3.08% K.

### 4.2. Experimental Design

The pot experiment was conducted using ceramic pots (35 cm in height and 25 cm in diameter). For each soil type, four biochar application levels were used: 0, 10, 20, and 30 g·kg^−1^ (based on dry soil weight). The treatments were designated as follows: albic soil (A, A1, A2, and A3), brown soil (B, B1, B2, and B3), and sandy soil (S, S1, S2, and S3), with A, B, and S serving as the controls without biochar. The experiment followed a randomized block design with three replicates per treatment. One month prior to soybean planting, the designated amount of biochar was thoroughly mixed with the soil and allowed to equilibrate. During the growth process of soybeans, no fertilizer was applied, and watering and other cultivation management practices were kept consistent across all treatments.

### 4.3. Analysis of Soil and Soybean Samples

Plant and soil samples were collected during the pod-setting stage (late September) and harvest season (early October). The samples from the pod-setting stage were used for the soil potassium content and soybean potassium content analyses. Air-dried soil samples were sieved through a 20-mesh sieve to measure the water-soluble, exchangeable, and non-exchangeable potassium levels using water extraction, ammonium acetate extraction, and hot nitric acid extraction, respectively, following the methods in [13]. The soil samples were sieved through a 100-mesh sieve and digested using the sodium hydroxide melting method. The plant samples were withered at 105 °C, dried at 80 °C, and digested using the sulfuric acid–hydrogen peroxide method. The potassium concentrations in all the extracts and digests were quantified using flame photometry (M410, Sherwood, Cambridge, UK).

The samples collected during the harvest season were used to determine the soil properties and soybean seed parameters. Soil bulk density was measured using the ring-knife method. Air-dried soil samples were sieved through 100-mesh sieves to analyze the total nitrogen and total phosphorus levels and 20-mesh sieves to analyze the available nitrogen and available phosphorus levels and pH. Total nitrogen levels were measured with an elemental analyzer (Variomax CN, ELEMENTAR, Langenselbold, Germany); total phosphorus levels were determined using sodium hydroxide digestion followed by spectrophotometry. The available nitrogen and phosphorus levels were analyzed using the alkaline hydrolysis diffusion method and sodium bicarbonate extraction method, respectively. Mature soybean plants were air-dried, and the yield components (grain count and dry weight of main stems and branches) were assessed.

### 4.4. Soil Potassium Adsorption Sites

The determination of potassium adsorption sites in the soils was conducted using a sequential extraction method combined with conventional techniques to quantify the different forms of potassium [39]. Specifically, 2.00 g of soil (mass m) was placed in a 25 mL centrifuge tube (initial mass m_0_), and 20.0 mL of distilled water was added. The mixture was shaken at 150 r/min for 30 min, centrifuged at 4000 r/min for 5 min, and the potassium concentration in the supernatant (c_1_) was measured. The supernatant was discarded, and the tube’s mass was recorded (m_1_). Next, 20 mL of 0.5 mol/L neutral CaCl_2_ was added to the sediment, followed by shaking, centrifugation, and measurement of the supernatant’s potassium concentration (c_2_). After discarding the supernatant and recording the mass (m_2_), 20.0 mL of 1 mol/L neutral NH_4_OAc was added, and the process was repeated to measure c_3_.

Acid-soluble potassium (Q_acid_) was determined using 2.50 g of soil and the 1 mol/L hot nitric acid method [13]. The contents of the different potassium fractions were calculated as follows:

Q_W_= c_1_v_1_/m

Q_e_ = (c_3_v_3_ − c_2_Δv_2_)/m

Q_i_ = Q_acid_ − Q_W_ − Q_p_ − Q_e_

Δv_1_ = (m_1_ − m)/ρ_1_, where ρ_1_ = 1.00 g/mL (distilled water density). Δv_2_ = (m_2_ − m)/ρ_2_, where ρ_2_ = 1.02 g/mL (CaCl_2_ solution density). Q_W_, Q_p_, Q_e_, and Q_i_ represent the water-soluble potassium, p-site potassium, e-site potassium, and i-site potassium contents, respectively.

### 4.5. Quantity–Intensity (Q/I) Isotherms

The quantity–intensity (Q/I) curve for soil potassium was determined using the shaking method outlined in [40]. Nine soil samples, each sieved through a 1 mm mesh and weighing 0.2, 0.3, 0.5, 1.0, 2.0, 3.0, 4.0, 5.0, and 6.0 g, respectively, were placed into individual 100 mL polypropylene centrifuge tubes. To each tube, 50 mL of a solution containing varying concentrations of KCl (0, 0.01, 0.05, 0.1, 0.2, 0.5, 1.0, 1.5, and 2.0 mmol·L^−1^) and a constant concentration of CaCl_2_ (2 mmol·L^−1^) was added.

The tubes were capped and shaken at a constant temperature of 25 °C at 150 rpm for 4 h and then allowed to stand for 24 h. Following this, the samples were centrifuged at 1500 rpm for 5 min, and the supernatant was filtered. The concentrations of potassium (K), calcium (Ca), and magnesium (Mg) in the equilibrium solutions were measured using an atomic absorption spectrophotometer.

Calculations:

The soil potassium Q/I curve was derived using the following approach. The activity coefficients (denoted as r) of the electrolytes KCl and CaCl_2_ in the equilibrium solution, along with the activity ratio of potassium ions (AR_k_), were calculated based on the Debye–Hückel theory using the equations from references [41,42]:

$$\log_{10}r\pm =\frac{-Az^{+}z^{-}\sqrt{I}}{1+\alpha \beta \sqrt{I}}$$

where r is the mean activity coefficient of the electrolyte; z represents the charges of the positive and negative ions; I is the ionic strength of the solution; A is a temperature-dependent constant (equal to 0.5090 at 25 °C); and α is a constant related to the ionic radius (equal to 1.9638 for CaCl_2_ and 0.9819 for KCl).

The ionic strength (I) was calculated using the following formula:

$$I=\frac{1}{2}\sum_{i}C_{i}Z_{i}^{2}$$

where C_i_ is the concentration of ion i and Z_i_ is the valence of ion i.

The activity ratio of potassium ions (AR_k_) in the equilibrium solution was determined using the following formula:

$${AR}_{0}=\frac{{C_{k}\left(r_{{CaCl}_{2}}\right)}^{2}}{\sqrt{\left(C_{Ca}+C_{Mg}\right){\left(r_{Ca{Cl}_{2}}\right)}^{3}}}$$

where C_K_, C_Ca_, and C_Mg_ represent the concentrations of K^+^, Ca^2+^, and Mg^2+^ in the equilibrium solution, respectively, and rKCl and rCaCl_2_ are the activity coefficients of KCl and CaCl_2_ in the equilibrium solution, respectively. The difference in potassium concentration in the solution before and after equilibrium (ΔK) was calculated as the difference in the potassium ion concentration in the initial solution and equilibrium solution. Then, the Q/I curve was plotted with ARₖ values on the horizontal axis and ΔK values on the vertical axis.

### 4.6. Statistical Analysis

The data were processed using Microsoft Excel 365. The statistical analyses were performed in SPSS 22, including one-way ANOVA to evaluate the soil properties, different forms of potassium, soybean potassium uptake, and potassium accumulation, followed by Duncan’s multiple range test for multiple comparisons. Correlation and variance analyses for soybean potassium accumulation, soil properties, and different potassium forms were conducted in SPSS 24.0, with significant differences between treatments determined using Tukey’s method (*p* < 0.05). Multivariate analyses were performed in R (4.3.3), including principal component analysis (PCA) using the vegan package, the Mantel test using the linkET package, Random Forest analysis using the Random Forest package (using rfUtilities and rfPermute for significance testing), and partial least squares path modeling using the plspm package. The goodness of fit (GOF) index was used to assess the model’s predictive performance.

## 5. Conclusions

This study elucidated the regulatory mechanisms through which biochar enhances soil potassium supply capacity and promotes crop potassium accumulation. Through an investigation of biochar’s effects on diverse soil types and soybean K dynamics, we demonstrated that biochar increases the soil available K content by introducing water-soluble K while simultaneously improving the soil’s long-term potassium buffering capacity through the redistribution of the e-site and i-site potassium fractions. Furthermore, biochar enhances soybean potassium accumulation through two pathways: direct mechanisms (e.g., potassium supplementation) and indirect mechanisms (e.g., soil structure optimization and nutrient retention). The biochar significantly improved the potassium supply capacity and crop potassium accumulation across all soil types, although the optimal application rate varied based on the soil type. Considering both the soil potassium supply capacity and crop potassium accumulation, the optimal biochar application rates were 20 g/kg for the albic soil, 10 g/kg for the sandy soil, and 20 g/kg for the brown soil. In summary, these findings underscore biochar’s dual functionality as both a potassium fertilizer and a soil conditioner, offering a sustainable strategy to alleviate potassium deficits in intensively managed agricultural systems.

## Figures and Tables

**Table 1:** Effect of different application rates of biochar (0, 10, 20, and 30 g/kg) on soil total and available nitrogen and phosphorous levels, pH, and bulk density.

	Total Nitrogen (g·kg^−1^)	Total Phosphorus (g·kg^−1^)	Available Nitrogen (mg·kg^−1^)	Available Phosphorus (mg·kg^−1^)	Soil pH	Bulk Density (g·cm^−3^)
Albic soil						
A	0.83 ± 0.01b	0.28 ± 0.03b	52.67 ± 1.15c	11.70 ± 2.40b	5.66 ± 0.24b	1.34 ± 0.01a
A1	0.87 ± 0.01a	0.39 ± 0.01a	57.00 ± 1.00a	14.57 ± 1.21a	6.53 ± 0.12a	1.28 ± 0.02b
A2	0.96 ± 0.01a	0.38 ± 0.01a	54.67 ± 0.58b	15.43 ± 1.62a	6.66 ± 0.41a	1.26 ± 0.07b
A3	0.99 ± 0.02a	0.38 ± 0.04a	58.67 ± 0.58a	12.77 ± 2.14b	6.92 ± 0.21a	1.16 ± 0.04c
Sandy soil						
S	0.32 ± 0.01b	0.09 ± 0.01b	27.67 ± 0.18b	13.57 ± 0.45a	7.96 ± 0.32a	1.55 ± 0.03a
S1	0.37 ± 0.01a	0.12 ± 0.01a	26.67 ± 0.23c	13.40 ± 0.69a	7.06 ± 0.28c	1.52 ± 0.02a
S2	0.4 ± 0.02a	0.11 ± 0.01a	28.33 ± 0.58a	13.40 ± 0.69a	7.30 ± 0.34b	1.45 ± 0.04b
S3	0.48 ± 0.02a	0.12 ± 0.00a	27.67 ± 0.41b	13.43 ± 1.11a	8.14 ± 0.13a	1.39 ± 0.08c
Brown soil						
B	1.15 ± 0.02b	0.52 ± 0.00b	87.00 ± 0.00b	21.43 ± 0.72b	7.30 ± 0.60b	1.25 ± 0.01a
B1	1.16 ± 0.01a	0.55 ± 0.00a	93.02 ± 0.21a	22.27 ± 1.42a	7.97 ± 0.25a	1.20 ± 0.01a
B2	1.26 ± 0.00a	0.58 ± 0.02a	80.00 ± 0.33c	22.50 ± 1.10a	8.17 ± 0.03a	1.13 ± 0.03b
B3	1.26 ± 0.00a	0.57 ± 0.01a	88.33 ± 0.58b	22.87 ± 1.16a	8.13 ± 0.01a	1.15 ± 0.02b

The different lowercase letters in the same column indicate significant differences (*p* < 0.05) between the different amounts of biochar applied to the same soil type.

**Table 2:** Effect of different application rates of biochar (0, 10, 20, and 30 g/kg) on soil available potassium, water-soluble potassium, exchangeable potassium, and non-exchangeable potassium contents.

	**Available** **Potassium** **(mg·kg^−1^)**	**Water-Soluble** **Potassium** **(mg·kg^−1^)**	**Exchangeable** **Potassium** **(mg·kg^−1^)**	**Non-Exchangeable** **Potassium** **(mg·kg^−1^)**
Albic soil				
A	367.24 ± 0.47d	15.21 ± 3.90b	48.71 ± 1.06d	303.33 ± 2.29b
A1	378.19 ± 6.94c	17.56 ± 2.40b	57.25 ± 1.60c	303.38 ± 12.73b
A2	427.55 ± 0.52b	19.80 ± 1.07b	66.69 ± 2.18b	341.06 ± 0.89a
A3	458.48 ± 8.17a	27.73 ± 2.89a	77.03 ± 1.78a	353.73 ± 15.77a
Sandy soil				
S	272.88 ± 0.11d	34.59 ± 3.13b	15.10 ± 2.84d	223.19 ± 0.45c
S1	289.72 ± 3.10c	35.16 ± 0.70b	24.79 ± 0.66c	229.78 ± 10.24b
S2	313.68 ± 0.60b	37.46 ± 1.70ab	49.01 ± 4.31b	227.22 ± 2.68bc
S3	439.08 ± 2.60a	47.75 ± 9.70a	78.60 ± 3.15a	312.73 ± 2.07a
Brown soil				
B	1269.42 ± 5.28d	17.36 ± 2.51b	56.87 ± 0.61b	1195.2 ± 10.63c
B1	1381.50 ± 8.18c	20.54 ± 4.99b	65.00 ± 3.59b	1295.96 ± 16.24b
B2	1421.76 ± 8.87b	23.93 ± 2.59b	83.79 ± 4.38a	1314.04 ± 18.01b
B3	1763.37 ± 12.12a	38.92 ± 6.84a	85.43 ± 5.29a	1639.02 ± 22.43a

The different lowercase letters in the same column indicate significant differences (*p* < 0.05) between the different amounts of biochar applied to the same soil type.

**Table 3:** Effect of different application rates of biochar (0, 10, 20, and 30 g/kg) on the amount of potassium absorbed (p-, e-, and i-sites) by soil.

p-Site Potassium (mg·kg^−1^)	e-Site Potassium (mg·kg^−1^)	i-Site Potassium (mg·kg^−1^)
Albic soil		
40.02 ± 2.24d	44.07 ± 1.08a	267.95 ± 2.12d
46.69 ± 3.60c	35.79 ± 0.67b	278.15 ± 3.79c
57.88 ± 2.57b	37.26 ± 2.08b	312.61 ± 0.46b
65.03 ± 2.05a	44.00 ± 2.34a	321.73 ± 2.67a
Sandy soil		
35.80 ± 1.66a	19.99 ± 0.93a	182.49 ± 3.75d
36.50 ± 1.66a	21.15 ± 1.71a	200.26 ± 3.73c
40.03 ± 0.63a	22.02 ± 5.54a	214.18 ± 5.70b
38.70 ± 3.13a	22.47 ± 1.04a	330.16 ± 5.79a
Brown soil		
47.80 ± 1.51b	57.66 ± 3.52b	1146.60 ± 4.00d
53.12 ± 3.18b	80.80 ± 3.93a	1227.04 ± 4.56c
71.89 ± 3.18a	84.09 ± 3.81a	1241.84 ± 2.43b
72.84 ± 0.66a	87.66 ± 4.92a	1563.95 ± 5.44a

The different lowercase letters in the same column indicate significant differences (*p* < 0.05) between the different amounts of biochar applied to the same soil type.

**Table 4:** Effect of different application rates of biochar (0, 10, 20, and 30 g/kg) on the soil potassium Q/I curve parameters.

	AR0	ΔK	Kl	Kx	PBC
Albic soil					
A	1.81	0.13	0.14	0.0096	70.38
A1	2.29	0.14	0.17	0.0246	62.13
A2	3.41	0.16	0.20	0.0358	48.40
A3	3.56	0.16	0.23	0.0687	46.00
Sandy soil					
S	1.29	0.04	0.21	0.1616	34.61
S1	2.09	0.06	0.20	0.1358	30.20
S2	3.06	0.07	0.21	0.1346	24.39
S3	3.07	0.07	0.28	0.2038	23.49
Brown soil					
B	1.91	0.13	0.17	0.0416	67.63
B1	2.18	0.16	0.2	0.0409	72.05
B2	2.54	0.18	0.23	0.0488	70.10
B3	2.68	0.17	0.29	0.1148	64.90

**Table 5:** Effect of different application rates of biochar (0, 10, 20, and 30 g/kg) on potassium absorption by soybean plants.

	Potassium Accumulation in Pods (mg·plant^−1^)	Potassium Accumulation in Stem (mg·plant^−1^)	Potassium Accumulation in Leaves (mg·plant^−1^)	Potassium Accumulation in Soybeans (mg·plant^−1^)
Albic soil				
A	192.38 ± 0.24d	42.99 ± 2.64d	54.35 ± 9.92c	289.72 ± 1.04d
A1	293.65 ± 3.44b	58.47 ± 3.44c	67.95 ± 3.12b	420.07 ± 0.56c
A2	313.71 ± 1.60a	71.54 ± 4.88b	74.63 ± 6.88a	459.88 ± 3.20a
A3	264.6 ± 0.24c	94.16 ± 4.4a	76.21 ± 1.20a	434.97 ± 4.32b
Sandy soil				
S	466.83 ± 4.64d	96.32 ± 5.12d	87.95 ± 4.64d	651.10 ± 3.84d
S1	654.72 ± 0.88b	244.28 ± 3.68b	155.68 ± 2.64c	1054.68 ± 2.8b
S2	510.35 ± 3.12c	206.36 ± 7.12c	163.01 ± 4.64b	879.72 ± 0.32c
S3	772.08 ± 9.2a	336.56 ± 4.24a	194.53 ± 4.4a	1303.17 ± 5.44a
Brown soil				
B	181.68 ± 4.24b	71.74 ± 5.20d	38.75 ± 1.20d	292.17 ± 2.48d
B1	127.03 ± 3.36c	99.94 ± 3.84c	84.88 ± 0.72c	311.85 ± 2.24c
B2	232.04 ± 0.88a	106.49 ± 2.32b	72.02 ± 7.04b	410.55 ± 1.12b
B3	187.84 ± 0.02b	144.77 ± 5.36a	95.39 ± 4.32a	428.01 ± 1.92a

The different lowercase letters in the same column indicate significant differences (*p* < 0.05) between the different amounts of biochar applied to the same soil type.

**Table 6:** Effect of different application rates of biochar (0, 10, 20, and 30 g/kg) on different yield components of soybean plants.

Treatment	Main Stem Seed Number (Seed·Plant^−1^)	Main Stem Seed Weight (g·Plant^−1^)	Branch Seed Number (Seed·Plant^−1^)	Branch Seed Weight (g·Plant^−1^)	Total Seed Weight (g·Plant^−1^)
Albic soil					
A	23.67 ± 1.25b	3.86 ± 0.48c	23.67 ± 2.36c	7.31 ± 0.22c	11.17 ± 0.47c
A1	34.00 ± 3.74a	7.97 ± 0.87a	45.33 ± 3.4b	8.92 ± 1.14b	16.88 ± 0.60b
A2	38.67 ± 2.87a	8.06 ± 0.74a	44.67 ± 3.3b	9.09 ± 1.43b	17.15 ± 2.04b
A3	29.00 ± 1.63b	6.71 ± 0.55b	55.33 ± 2.87a	14.62 ± 1.49a	21.33 ± 1.91a
Sandy soil					
S	23.33 ± 2.05d	4.92 ± 0.89d	41.00 ± 1.63b	9.45 ± 0.66b	14.37 ± 1.37d
S1	47.67 ± 1.25b	9.92 ± 0.39b	54.00 ± 0.82a	11.20 ± 0.26a	21.12 ± 0.65a
S2	43.00 ± 0.82c	10.40 ± 0.18c	56.33 ± 4.50a	10.41 ± 1.13a	20.81 ± 1.31b
S3	54.33 ± 1.25a	11.07 ± 0.17a	43.33 ± 2.49b	7.71 ± 0.77c	18.78 ± 0.63c
Brown soil					
B	29.00 ± 1.63c	6.90 ± 0.38c	42.33 ± 2.05b	9.68 ± 0.87b	16.58 ± 1.23b
B1	37.67 ± 2.05b	8.93 ± 0.65b	50.33 ± 2.87a	12.81 ± 1.37a	21.74 ± 1.68a
B2	46.00 ± 3.56a	11.48 ± 1.03a	36.00 ± 2.45c	9.24 ± 0.16b	20.71 ± 1.18a
B3	32.33 ± 1.70b	5.56 ± 0.76c	24.00 ± 2.16d	4.12 ± 0.35c	9.67 ± 0.41c

The different lowercase letters in the same column indicate significant differences (*p* < 0.05) between the different amounts of biochar applied to the same soil type.

## Data Availability

The data are available on request to the corresponding author.
